# A Gender Factor in Shaping T-Cell Immunity to Melanoma

**DOI:** 10.3389/fonc.2015.00008

**Published:** 2015-02-02

**Authors:** Roxana S. Dronca, Haidong Dong

**Affiliations:** ^1^Division of Medical Oncology, College of Medicine, Mayo Clinic, Rochester, MN, USA; ^2^Department of Urology, College of Medicine, Mayo Clinic, Rochester, MN, USA; ^3^Department of Immunology, College of Medicine, Mayo Clinic, Rochester, MN, USA

**Keywords:** melanoma, immunity, T-cell, sex, gender identity

We have read with great interest the article by Wesa and colleagues in *Frontiers in Oncology* (1) describing the anti-tumor immune dysfunction in patients with melanoma and renal cell carcinoma. Accumulating evidence suggests that, in patients with cancer, the state of systemic immunity is not “normal”; rather, it is repolarized toward a state of Th-2 biased “chronic inflammation,” leading to suppression of cytotoxic CD8 T-cell function (2, 3). In this publication, the authors show that, in addition to this functional deficiency, tumor-associated antigen (TAA)-specific (but not total or viral specific) CD4+ T-cells are prone to express a pro-apoptotic phenotype in patients with active cancer. Similarly to previous reports, they show that there is evidence of immune activation in the peripheral blood of patients with advanced malignancies, manifested by increased frequencies of tumor-specific CD4+ T-cells in patients with active disease as opposed to patients rendered no evidence of disease (NED); however, these cells seems to have an enhanced sensitivity to activation-induced cell death (AICD) via an apoptotic mechanism. Interestingly, they found that female patients with melanoma had significantly higher frequencies of TAA-specific T-cells as compared to male patients, raising the possibility of sex differences in anti-tumor immunity in this disease. This is very intriguing as malignant melanoma has not classically been viewed as a hormone-sensitive neoplasm; however, an accumulating body of evidence suggests that the outcome of established melanoma is influenced by endocrine status (4–6). Epidemiological studies have consistently shown that males have poorer survival rates when compared to females. Specifically, men seem to present with prognostically worse primary tumors (thicker primary melanomas with a higher incidence of ulceration), that they have a higher probability of developing metastases, and that they experience a shorter survival compared with women (4, 5). Possible reasons for these differences were thought to be due to differences in detection (7, 8), given that males are reportedly less likely to self-detect their melanomas (7), make fewer visits to health-care providers, and are less likely to engage in preventive behaviors (8). However, gender seems to remain an independent prognostic indicator even after adjustment for these factors; additionally, when disease progression takes place, women seem to progress more frequently to local sites in the form of satellite or in-transit metastases, while men exhibit more frequently direct regional lymph node metastases or show further progression to distant sites (4). It has therefore been suggested that these differences may be due to estrogen status, as data suggest that the female survival advantage may not persist after menopause (5, 6). We have recently analyzed data from the National Cancer Institute surveillance, epidemiology, and end results (SEER) program, including 87,165 cases of primary invasive melanoma diagnosed between 1992 and 2009 and similarly found that melanoma-specific survival was significantly poorer for males as compared to females for localized and regional disease for all age groups, suggesting that sex may influence local and regional cancer progression (9). Therefore, we postulate that a biological basis may be responsible for the consistently observed differences in clinical outcomes in patients with malignant melanoma, such as the existence of sex differences in immunity.

The sexual dimorphism in the immune system of female and male patients has long been recognized by the existence of sex differences in the incidence and course of autoimmune diseases (10, 11). It has been recently postulated that these immune differences could also be of relevance to the natural course and surviving chronic inflammatory conditions such as cancer. Nevertheless, the molecular differences for the sex-based differences in the outcome of malignant melanoma and other malignancies remain undefined, and, as of yet, the mechanism of this apparent female survival benefit has neither been investigated in any great depth nor have its implications been exploited with respect to therapy. In their article, Wesa et al. (1) speculate that the increased number and/or improved function of tumor-specific T-helper (Th) cells could serve as a foundation for exploring and understanding these clinical observations. Th1 cells can mediate the anti-tumor effects through a variety of mechanisms (12) and are therefore important therapeutic targets. In recent years, the Programed cell death 1 (PD-1) pathway has been found to play an important role in tumor-induced immune suppression (13) in melanoma and is an increasingly exploited therapeutic target in this disease and other advanced malignancies (14–16). While a role for sex-hormone modulation of PD-1 has emerged, the published literature is limited to animal studies and offers conflicting findings. For instance, preclinical studies suggest that the expression and function of PD-1 are responsive to sex steroids, and that the hormone-mediated effects on PD-1 signaling pathway play important roles in mediating autoimmunity (17–19). The expression of the PD-1 ligand, PD-L1, or B7-homolog 1 (B7-H1) has also been shown to be modulated in an estrogen-dependent and sex-dependent manner (20). One recent study examined B7-H1 expression on regulatory T-cells (Treg) in B-16 melanoma bearing mice. Interestingly, despite comparable B7-H1 expression, female mice were more sensitive to PD-L1 blockade, and treatment with an anti-B7-H1 blocking monoclonal antibody reduced tumor growth to a greater degree in females compared to male mice, as a result of greater reduction in Treg function and increase in tumor-specific cytotoxic (CD8) T-cells in females (20). The reasons for this differential PD-1/B7-H1 signaling in male versus female mice are not known. Notably, there are few studies published regarding sex-hormone modulation of PD-1/B7-H1 pathway in cancer, and work of this nature in humans is non-existent, despite accumulating evidence that this pathway plays a pivotal role in tumor-induced immune suppression (21). We believe that identification of sex-dependent differences in immune regulatory pathways important for immune evasion in melanoma and other advanced cancers will address an important question – whether males and females respond differently to certain immune therapies – a fact not incorporated into most clinical trial designs. Despite early successes with novel immune agents, preclinical models indicate that combinatorial therapies are likely to deliver maximum clinical impact; therefore, elucidation of the mechanisms responsible for these sex-based immune differences may identify novel treatment strategies for improving the protective aspects of T-cell immunity in malignant melanoma and other advanced malignancies.

## Conflict of Interest Statement

The authors declare that the research was conducted in the absence of any commercial or financial relationships that could be construed as a potential conflict of interest.
